# Curcumin Ameliorates Cisplatin-Induced Nephrotoxicity and Potentiates Its Anticancer Activity in SD Rats: Potential Role of Curcumin in Breast Cancer Chemotherapy

**DOI:** 10.3389/fphar.2017.00132

**Published:** 2017-04-04

**Authors:** Parveen Kumar, Chandana C. Barua, Kunjbihari Sulakhiya, Rajeev Kumar Sharma

**Affiliations:** ^1^Laboratory of Molecular Pharmacology and Toxicology, Department of Pharmacology and Toxicology, National Institute of Pharmaceutical Education and Research, GMCHGuwahati, India; ^2^Department of Pharmacology and Toxicology, College of Veterinary ScienceGuwahati, India; ^3^Department of Microbiology, College of Veterinary ScienceGuwahati, India

**Keywords:** brain-derived neurotrophic factor, breast cancer, cisplatin, curcumin, nephrotoxicity, peroxisome proliferator activated receptor-γ

## Abstract

Breast malignant neoplastic disease is one of the most complex diseases, as it is a multifactorial disease in which virtually all the targets are instantly or indirectly inter-reliant on each other. Cisplatin (CIS), an inorganic antineoplastic agent is widely utilized in the treatment of various solid tumors including breast cancer. Despite everything, its clinical use is limited, due to ototoxicity, peripheral neuropathy, and nephrotoxicity. The present work was directed to assess the combined result of curcumin (CUR) and CIS in 7, 12-dimethyl benz[a]anthracene (DMBA) induced breast cancer in rats and the prevention of nephrotoxicity induced by the latter. CIS-induced nephrotoxicity was assessed by change in body weight, kidney weight, altered levels of BUN, creatinine, TNF-α, IL-6, IL-8, IL-10, and histopathology of the kidney. Anticancer activity was assessed by measurement of tumor weight, tumor volume, % tumor inhibition, levels of PPAR-γ, and BDNF in mammary tumors and histopathology of mammary tumors. CUR pre-treatment mitigated nephrotoxicity by reducing the inflammatory markers (TNF-α, IL-6, and IL-8; *p* < 0.001). Further, it reduced mammary cancer via increasing the expression of PPAR-γ (*p* < 0.001) and decreasing the expression of BDNF (*p* < 0.001) in mammary tumors. It also reduced tumor volume, further postulating that CUR might adjunct the anticancer activity of the CIS. To the best of our knowledge, this is the first report, which showed that CUR ameliorated CIS-induced nephrotoxicity and improved its anticancer activity in DMBA induced breast cancer in female Sprague-Dawley rats.

## Introduction

The prevalence of breast carcinoma is increasing by leaps and bounds especially in the developing countries (Kumar et al., 2013b). It is the most prevalent form of cancer in developed countries and the second most commonly diagnosed malignancy in the third world countries (Kumar et al., 2015). Increased incidence of breast cancer in females; especially the younger ones, demands prompts, and intense interventions making the therapy more effective and less toxic (Kumar et al., 2013a; Jamdade et al., 2015b).

Cisplatin (CIS), a frequently employed broad-spectrum antineoplastic agent, remains to be a preferred treatment modality for various malignancies despite ototoxicity, peripheral neuropathy, and nephrotoxicity (Mundhe et al., 2015; Jamdade et al., 2015a). The complex spectrum of CIS nephrotoxicity includes DNA damage, tubular toxicity, and inflammation (Kumar et al., 2013c). The inflammation plays a central pathophysiological role in CIS associated nephrotoxicity, as CIS injection induces a cascade of inflammatory responses in the kidney through the release of several cytokines (TNF-α, IL-1β etc.) and chemokines [MCP-1, macrophage inflammatory protein (MIP)-2 etc.]. TNF-α plays a significant role in the production of other inflammatory cytokines and chemokines and is a chief wrongdoer of the CIS-induced inflammatory renal injury (Deng et al., 2001; Ramesh and Reeves, 2002; Kumar et al., 2013c). Times and again, umpteen theories have been proposed for prevention of its nephrotoxicity but of little/no avail (Ueki et al., 2013).

Curcumin (CUR), 1,7-bis(4-hydroxy 3-methoxy phenyl)-1,6-heptadione-3,5-dione or diferuloylmethane is a natural yellow-colored polyphenol derived from the perennial herb *Curcuma longa*, commonly called turmeric. The three major ingredients of commercial CUR are: curcumin (77%), demethoxycurcumin (17%), and bisdemethoxycurcumin (3%) together referred to as curcuminoids (Aggarwal et al., 2003; Agrawal and Mishra, 2010). The different biological and pharmacological actions of CUR e.g., anti-inflammatory, antioxidant, anti-ischemic, antibacterial, antifungal, and anticancer are due to different methoxy substitutions in the chemical structure of these compounds (Nabavi et al., 2014). Curcumin (CUR) can diminish renal damage by modulating organic anion export markers, drug resistance markers, through suppression of mTOR effector pathways or inhibition of NF-κB, TNF-α, IL-6 etc. (Jobin et al., 1999; Kuhad et al., 2007; Ueki et al., 2013). The combination of CUR and α-tocopherol is renoprotective by inhibiting of NADPH oxidase (Palipoch et al., 2013). Former studies have urged that usage of rosiglitazone and CUR (anti-inflammatory agents) is safe and one of the key approaches to attenuate CIS-induced renotoxicity (Ueki et al., 2013; Kumar et al., 2013c).

Peroxisome proliferator-activated receptor (PPAR)-γ belongs to the nuclear receptor superfamily of ligand-activated transcription factors. It heterodimerizes with the retinoid X receptor (RXR) and binds to the PPAR response element (PPRE; Yamaguchi et al., 2006). The ligands for PPAR-γ include synthetic agents like rosiglitazone, pioglitazone, and natural compound such as CUR (Jacob et al., 2007). PPAR-γ is mainly linked to differentiation of adipose tissue but it has likewise been reported to control the development, differentiation, and gene expression of different cancer cells (Barak et al., 1999; Gupta and Dubois, 2002). The agents like CUR can sensitize cancer cells to the cytotoxic action of chemotherapy, thereby cutting down the dosage and hence, the associated toxicities. Curcumin augments the anticancer effects of CIS and exerts its own anticancer activity by blocking transformation, tumor initiation, tumor promotion, invasion, angiogenesis, and metastasis (Aggarwal et al., 2003).

Brain-derived neurotrophic factor (BDNF) is a member of the nerve growth factor family and plays an important role in the survival and growth of neurones. Tropomyosin-related kinase B (TrkB) is the primary receptor of BDNF, which functions as a tyrosine kinase (Descamps et al., 2001; Blasco-Gutierrez et al., 2007). BDNF has been associated with several human neoplasms including ovarian, lung, prostate, hepatocellular, pancreatic, head and neck squamous cell carcinomas, and breast cancer (Patani et al., 2011). Interestingly, this nerve growth factor (NGF) has been demonstrated to stimulate proliferation, angiogenesis, and behaves as an anti-apoptotic factor in human breast cancer (Dolle et al., 2004; Adriaenssens et al., 2008).

Based upon the above facts, we hypothesize that pre-treatment of CUR along with CIS may diminish its nephrotoxicity and synergize its anticancer activity in chemically induced breast cancer in female Sprague-Dawley rats. In the current study, we examined the mechanism underlying the effect of CUR on CIS-induced renal damage and its antineoplastic efficacy.

## Methods

### Materials

DMBA, CIS, and CUR were purchased from Sigma (St. Louis, MO, USA). Interleukin (IL)-6, IL-8, IL-10, and tumor necrosis factor (TNF)-α enzyme-linked immunosorbent assay (ELISA) kits were purchased from Invitrogen (Invitrogen Corporation, Frederick, USA). The BDNF ELISA kit was purchased from Abnova (Abnova, Taipei, Taiwan). CIS solution was prepared in normal saline and CUR was suspended in carboxymethyl cellulose (CMC). The drug/molecular target nomenclature conforms to the BJP's Concise Guide to Pharmacology (Alexander et al., 2015). All the solutions were prepared fresh before each experiment.

### Animals

The experiments were performed on female Sprague-Dawley rats (National Institute of Nutrition, Hyderabad, India) maintained at the Animal House in the Department of Pharmacology, Guwahati Medical College and Hospital (GMCH) Assam. The rats were put up and maintained at temperature and humidity levels as defined in the Guide for the Care and Use of Laboratory Animals, Public Health Service Policy on Humane Care and Use of Laboratory Animals. The work was sanctioned by the GMCH Institutional Animal Ethics Committee (approval number: MC/32/2013/2) and all experiments were taken in accordance with the Guide for the Care and Use of Laboratory Animals, Public Health Service Policy on Humane Care and Use of Laboratory Animals, and Animal Welfare Act. All studies involving animals are reported in accordance with the ARRIVE guidelines for reporting experiments involving animals (McGrath et al., 2010). Standard animal feed (Pranaw Agro Industries, New Delhi) and water were provided to the animals *ad libitum*. The chemically induced mammary tumor animals were examined daily for signs of distress or bother. Extra care was given at 12th week when the tumors were developed. The overall clinical status, including appearance, attitude, body temperature, presence of persistence anorexia, and/or labored respiration, behavioral and physiological reactions of every tumor bearing animals were routinely monitored. The food and water intake, body weight, and tumor volume were assessed frequently. The animals were checked for ulceration or distension of tumors. Rats which were expected soon to become moribund were anaesthetized and killed humanely by cervical dislocation.

### Tumor induction

Female Sprague-Dawley rats at the age of 8 weeks weighing 160–180 g were gavaged with DMBA (60 mg/kg body weight), a dose sufficient to make 100% tumor incidence in the control group over the course of the study (Whitsett et al., 2006; Tikoo et al., 2009a). The DMBA was dissolved in olive oil in a stock solution of 30 mg/ml.

### Experimental design

**Figure 1:**
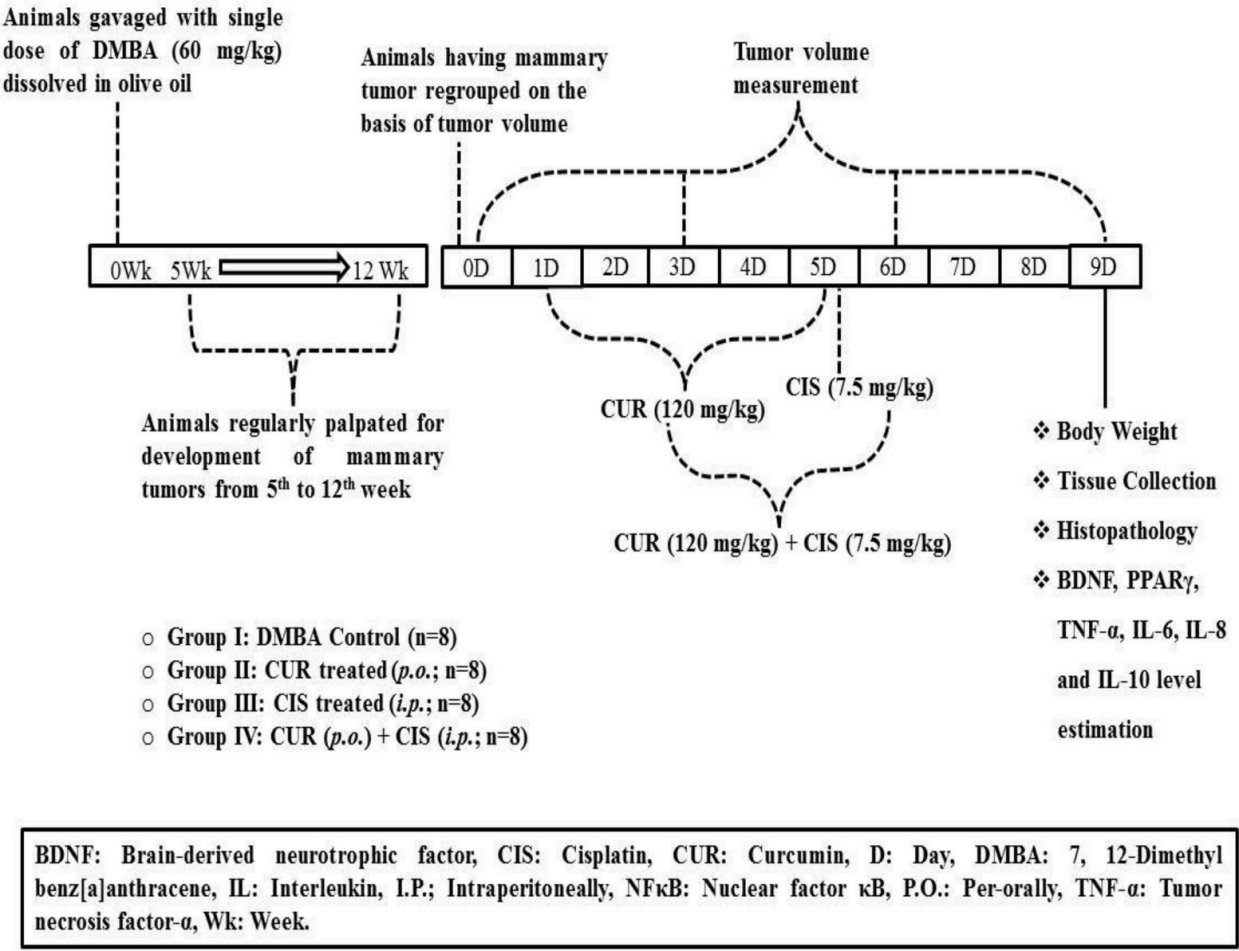
**Timeline of the study**.

Initially, 40 animals were administered with DMBA, out of which 5 animals died within 5 weeks of DMBA administration. Further, 2 more animals died during the tumor development period; yet, the death was imputed to the mammary tumors. Animals were palpated twice a week, starting 5 weeks after DMBA administration in order to tape the visual aspect, position and size of tumors. The mammary tumors reached measurable level after 12 weeks of DMBA administration. One animal was not included while grouping since it was harboring disproportionately grown mammary tumors. The animals were sacrificed when the tumor diameter reached 3 cm, animals became moribund (Exclusion criteria), or after the culmination of the experimentation. After 12 weeks, DMBA treated rats were grouped into 4 different groups on the basis of their tumor volume. DMBA treated rats received normal saline (Group I). Breast cancer-induced rats were treated with CUR (120 mg/kg) suspended in 0.25% w/v CMC through oral gavage for 5 days (Group II). Breast cancer-induced rats were treated with CIS (7.5 mg/kg) dissolved in normal saline (0.9% w/v) by intraperitoneal route (Group III). Breast cancer-induced rats were treated with CUR (120 mg/kg) suspended in 0.25% w/v CMC for 5 days followed by a single dose of CIS dissolved in normal saline (0.9% w/v) by intraperitoneal route on the 5th day was assigned as Group IV (Figure 1). Rats were weighed prior to the injection and 4 days after CIS treatment. Blood samples were collected on the 5th day from the rat tail veins under light ether anesthesia in heparinized centrifuge tubes and plasma was separated by centrifugation at 2,300 g. Plasma was stored at −80°C until assayed. The study design and animal ethics conform to the recent guidance on experimental design and analysis (Curtis et al., 2015). All the animals were maintained on a standard diet and water during the entire period of study.

### Measurement of tumor volume and % tumor inhibition

The measurements were done for visible tumors; two diameters i.e., shortest and longest diameter of the tumors was measured. The volume of the tumor was calculated as π/6 (a)^2^*(b), where a is the smallest and b is the longest length of the tumor. Percentage tumor inhibition was calculated by taking tumor volume of day 0 of all the groups as 100% and then percentage inhibition was calculated by comparing 0th day tumor volume with 3rd, 6th, and 9th day tumor volumes of the respective groups.

**Table:** 

Group	Animal number	Shortest diameter (a)	Longest diameter (b)	Formula (= π/6^*^a^2*^b)	Tumor Volume (mm^3^)
Breast Cancer Control (BCC)	1	0.33 cm	0.44 cm	= 3.14/6^*^0.33^2*^0.44	24.92
				= 0.52^*^0.1089^*^0.44	
				= 0.02492 cm^3^	
				= 0.02492^*^1000 mm^3^	
				= 24.92 mm^3^	

### Estimation of blood urea nitrogen, creatinine, and plasma albumin

Blood samples were collected in heparinized centrifuge tubes and immediately centrifuged at 2,300 g for the separation of plasma and were stored at −80°C until assayed. The plasma was used for the estimation of blood urea nitrogen (BUN), creatinine and albumin as described previously (Kumar et al., 2013c).

### Measurement of inflammatory markers and BDNF level

The levels of TNF-α, IL-6, IL-8, and IL-10 in breast cancer tissue and BDNF in plasma were determined by using ELISA kits, according to the manufacturer's instructions. In all the cases, a standard curve was constructed from the criteria provided by the producer.

### Histopathology of kidney and mammary tumor

Histopathology of the kidney and the mammary tumor was performed as described previously (Mundhe et al., 2015; Jamdade et al., 2015a). Briefly, for light microscopy, autopsy samples were taken from the kidney and the mammary tumor of rats from different groups and fixed in 10% formal saline for 24 h. The washing was made out with distilled water, then serial dilutions of alcohol (methyl, ethyl, and absolute ethyl) were used for drying up. Specimens were cleared in xylene and embedded in paraffin at 56°C in hot air oven for 24 h. Paraffin beeswax tissue blocks were prepared for sectioning at a thickness of 4 μm by sledge microtome. The obtained tissue sections were collected on glass slides, deparaffinized, stained with haematoxylin and eosin stain for routine examination then examination was done through the light electric microscope.

### Protein isolation and western blotting

Mammary gland tumor (~50 mg) was homogenized with ice-cold 500 ml mixture of radioimmunoprecipitation assay (RIPA) buffer and protease inhibitor cocktail. The homogenate was centrifuged (13,000 g, 20 min, 4°C) and the supernatant containing protein was collected and stored at −80°C. Protein concentration in the supernatant was determined using the method of Lowry et al. (1951). For western blot analysis, SDS-PAGE was carried out utilizing a vertical midi gel system (GeneiTM, Merck). Briefly, 10–20 μg of protein sample was mixed with the appropriate measure of a freshly prepared Laemli buffer. The mix was heated at 70°C for 10 min on a water bath; loaded onto the polyacrylamide gel and run at 100 V for around 120 min. Protein was transferred onto a nitrocellulose membrane using semi-dry blotter (Merck Millipore) at 90 mA for 1 h. Immunoblot analysis was performed by using Western DotTM 625 Goat Anti-Rabbit Western Blot Kit using the supplier's manual. Following primary antibodies were used: monoclonal anti-β-actin antibody, 1:2,000 (Sigma-Aldrich); an antibody to BDNF (rabbit 1:1,000, Santa Cruz, CA) and antibody to PPAR-γ (rabbit 1:2000, Santa Cruz, CA). The membrane was imaged using a UV transilluminator (Biostep UST-20M-8E & ArgusX1). Quantitative analysis of the picture was done using ImageJ software. The results were normalized with respect to β-actin.

### Statistical analysis

Data are represented as means ± S.E.M. and were analyzed by Prism 5.0 statistical program (GraphPad Software Inc., San Diego, CA, USA). Comparisons between experimental groups were performed using one-way ANOVA followed by Tukey's *post-hoc* test. Differences were considered significant if the *p-*value was less than 0.05.

## Results

### Combined effect of CUR and CIS on body weight, kidney weight, and tumor weight

**Figure 2:**
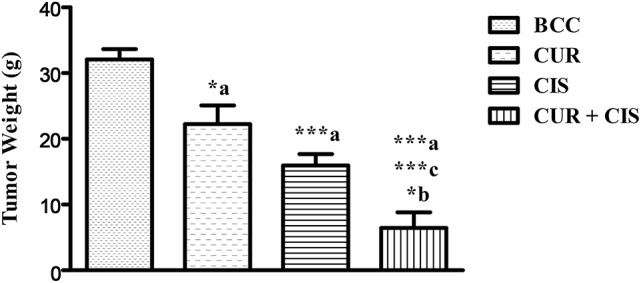
**Combined effect of curcumin and cisplatin on tumor weight in breast cancer rats**. All the values were expressed as mean ± SEM (*n* = 8). ^*^P < 0.05, ^***^P < 0.001. a vs. breast cancer control, b vs. cisplatin, and c vs. curcumin. Where BCC is breast cancer control, CUR is curcumin, CIS is cisplatin, and CUR + CIS is pre-treatment of curcumin (120 mg/kg) for 5 days, followed by single dose of cisplatin (7.5 mg/kg) on the 5th day.

Body weight, kidney weight and tumor weight were assessed at the end of the study. As shown in Table 1, CIS treated rats showed a significant loss in body weight when compared with mammary cancer control rats. There was no significant change in the body weight of curcumin treated rats when compared with mammary cancer control rats. CUR pre-treatment for 5 days in CIS treated rats showed a significant gain in body weight (*p* < 0.001) as compared to CIS treated mammary cancer rats. Moreover, substantial growth in kidney weight (*p* < 0.001) was observed in CIS treated rats, as compared to mammary cancer control rats. CUR pre-treatment for 5 days restored the kidney weight (*p* < 0.001) to mammary cancer control rats' kidney weight. Mammary cancer control rats presented maximum tumor weight as compared to drug treated groups. Treated animals showed a substantial decrease in the tumor weights as compared to cancer control animals. Furthermore, CUR plus CIS combination treated rats exhibited a significant decrease (*p* < 0.001) in the tumor weight as compared to CUR (*p* < 0.001) and CIS (*p* < 0.05) alone treated mammary cancer rats (Figure 2).

**Table 1:** **Effect of curcumin plus cisplatin treatment on body weight, kidney weight, BUN, creatinine, and albumin**.

	**Body weight (g)**	**Kidney weight (g)**	**BUN (mg/dl)**	**Creatinine (mg/dl)**	**Albumin (g/dl)**
BCC	238 ± 2.2	0.601 ± 0.02	21 ± 3.53	1.0 ± 0.12	3.99 ± 0.12
CUR	248 ± 3.1	0.610 ± 0.03	23 ± 2.85	1.03 ± 0.05	3.91 ± 0.09
CIS	218 ± 2.6^***^^a^	0.798 ± 0.02^***^^a^	149 ± 8.20^***^^a^	3.07 ± 0.35^***^^a^	2.23 ± 0.14^***^^a^
CUR + CIS	235 ± 2.1^***^^b^	0.641 ± 0.01^***^^b^	52 ± 5.41^***^^b^	1.3 ± 0.23^***^^b^	3.74 ± 0.20^***^^b^

All the values were expressed as mean ± SEM (n = 8).

*** P < 0.001.

a vs. breast cancer control,

b *vs. cisplatin. Where BCC is breast cancer control, CUR is curcumin, CIS is cisplatin, and CUR + CIS is pre-treatment of curcumin (120 mg/kg) for 5 days, followed by single dose of cisplatin (7.5 mg/kg) on the 5th day*.

### Combined effect of CUR and CIS on renal function

Treatment of CIS showed a significant increase in the level of BUN (*p* < 0.001) when compared with mammary cancer control rats. CUR pre-treatment for 5 days followed by CIS treatment showed significant reduction in BUN (*p* < 0.001) level, as compared to CIS alone treated mammary cancer rats (Table 1). Treatment with CIS produced significant elevations of creatinine level (*p* < 0.001) when compared with mammary cancer control rats. Pre-treatment with CUR for 5 days before CIS treatment showed significant reduction of creatinine level (*p* < 0.01), as compared to CIS treated rats (Table 1). Furthermore, treatment of CIS significantly decreased plasma albumin levels (*p* < 0.001) as compared to cancer control animals. CIS injection damages the glomeruli by an inflammatory mechanism which results in the increased permeability of the glomerulus and podocytes (highly specialized cells) and this is responsible for the reduced level of albumin in the blood. Pre-treatment with CUR for 5 days prior to CIS treatment showed significant elevation of plasma albumin levels (*p* < 0.001), as compared to CIS treated breast cancer rats (Table 1). CUR alone treated rats exhibited no substantial change in BUN, creatinine, and albumin levels. These observations demonstrated that CUR pre-treatment was efficacious in reducing CIS-induced kidney injury in DMBA induced mammary carcinoma in female Sprague-Dawley rats.

### Combined effect of CUR and CIS on inflammatory markers in renal tissue in mammary cancer

**Figure 3:**
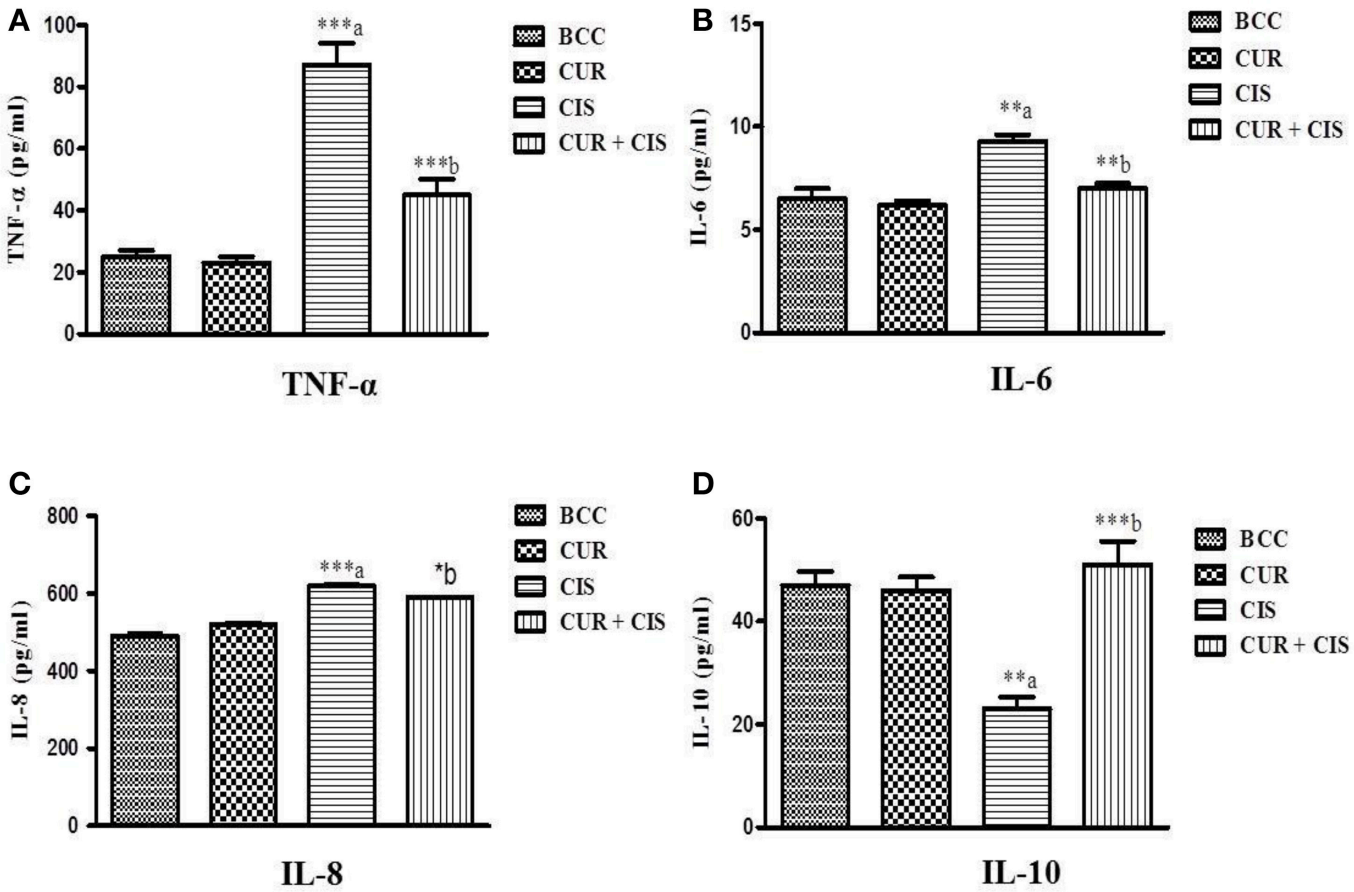
**Effect of curcumin plus cisplatin treatment on inflammatory markers in breast cancer (A–D)**. All the values were expressed as mean ± SEM (*n* = 8). ^*^P < 0.05, ^**^P < 0.01, ^***^P < 0.001. a vs. breast cancer control, b vs. cisplatin. Where BCC is breast cancer control, CUR is curcumin, CIS is cisplatin, and CUR + CIS is pre-treatment of curcumin (120 mg/kg) for 5 days, followed by single dose of cisplatin (7.5 mg/kg) on the 5th day.

Inflammatory markers were measured in the renal tissue at the end of the study in all the groups. Cisplatin-treated rats exhibited significantly increased levels of TNF-α (*p* < 0.001), IL-6 (*p* < 0.01), and IL-8 (*p* < 0.001) whereas, significantly decreased the level of IL-10 (*p* < 0.01) on the 5th day when compared with breast cancer control rats. However, curcumin pre-treatment for 5 days in cisplatin-treated rats significantly reduced the levels of TNF-α (*p* < 0.001), IL-6 (*p* < 0.01), and IL-8 (*p* < 0.05) whereas significantly improved the level of IL-10 (*p* < 0.001) as compared to cisplatin-treated breast cancer rats (Figure 3).

### CUR improves antitumor activity of CIS in mammary cancer

#### Tumor volume

**Figure 4:**
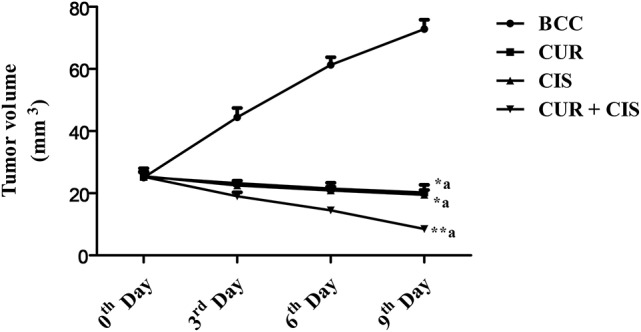
**Combined anticancer effect of curcumin pre-treatment and cisplatin on tumor volume (mm^3^) in breast cancer rats**. All the values were expressed as mean ± SEM (*n* = 8). ^*^P < 0.05, ^**^P < 0.01. a vs. breast cancer control. Where BCC is breast cancer control, CUR is curcumin, CIS is cisplatin, and CUR + CIS is pre-treatment of curcumin (120 mg/kg) for 5 days, followed by single dose of cisplatin (7.5 mg/kg) on the 5th day.

Figure 4 shows the tumor volumes of CUR, CIS, CUR plus CIS, and mammary cancer control rats. There was a considerable increase in tumor volume of mammary cancer control rats when compared with the drug treated rats, viz. CIS alone, CUR alone and CUR plus CIS treated rats. CIS (*p* < 0.05), CUR (*p* < 0.05), and CUR pre-treated (*p* < 0.01) rats showed a significant reduction in their tumor volumes when compared with mammary cancer control rats. However, CUR pre-treatment for 5 days followed by a single dose of CIS treated rats exhibited a maximum reduction (*p* < 0.01) in tumor volume when compared with CUR and CIS alone treated rats.

#### % Tumor inhibition

Table 2, shows the % tumor inhibition of treated (CUR, CIS, and CUR plus CIS) and mammary cancer control rats. There was a considerable tumor progression in breast cancer control rats as compared to CUR, CIS, and CUR plus CIS treated rats. In drug-treated rats, the tumor did not break off totally, but a substantial regression was recorded when compared with breast cancer control rats. CUR treated group showed 7% reduction on 3rd day, 15% reduction on the 6th day, and 20% on the 9th day of the tumor volume when compared with 0 day tumor volume. CIS treated rats showed 12% reduction on the 3rd day, 18% reduction on the 6th day, and 24% on the 9th day of the tumor volume when compared with 0 day tumor volume. In event of pre-treated rats, there was a 25% reduction on 3rd day, 43% reduction on the 6th day, and 66% reduction on the 9th day of the tumor volume when compared with 0 day tumor volume. From the above results, it was very clear that CUR pre-treatment (followed by CIS) treated rats showed more anticancer activity when compared with CIS and CUR alone treated rats.

**Table 2:** **The combined anticancer effects of curcumin and cisplatin on % tumor inhibition in breast cancer rats**.

	**0th Day**	**3rd Day**	**6th Day**	**9th Day**
BCC	100	178 (+78)	245 (+145)	292 (+192)
CUR	100	93 (−7)	85 (−15)	80 (−20)
CIS	100	88 (−12)	82 (−18)	76 (−24)
CUR + CIS	100	75 (−25)	57 (−43)	34 (−66)

*Where BCC is breast cancer control, CUR is curcumin, CIS is cisplatin, and CUR + CIS is pre-treatment of curcumin (120 mg/kg) for 5 days, followed by single dose of cisplatin (7.5 mg/kg) on the 5th day*.

### Combined effect of CUR and CIS on BDNF expression in mammary cancer rats

**Figure 5:**
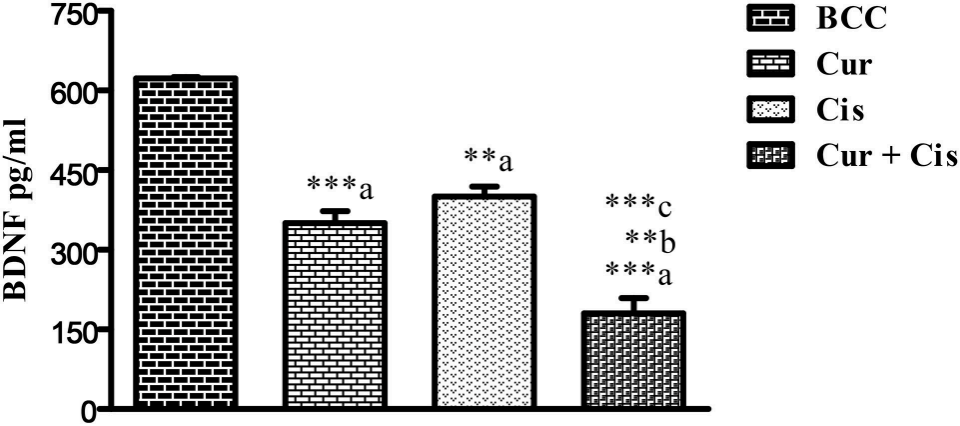
**Effect of curcumin plus cisplatin treatment on BDNF in breast cancer rats**. All the values were expressed as mean ± SEM (*n* = 8). ^**^P < 0.01, ^***^P < 0.001. a vs. breast cancer control, b vs. cisplatin, and c vs. curcumin. Where BCC is breast cancer control, CUR is curcumin, CIS is cisplatin, and CUR + CIS is pre-treatment of curcumin (120 mg/kg) for 5 days, followed by single dose of cisplatin (7.5 mg/kg) on the 5th day.

**Figure 6:**
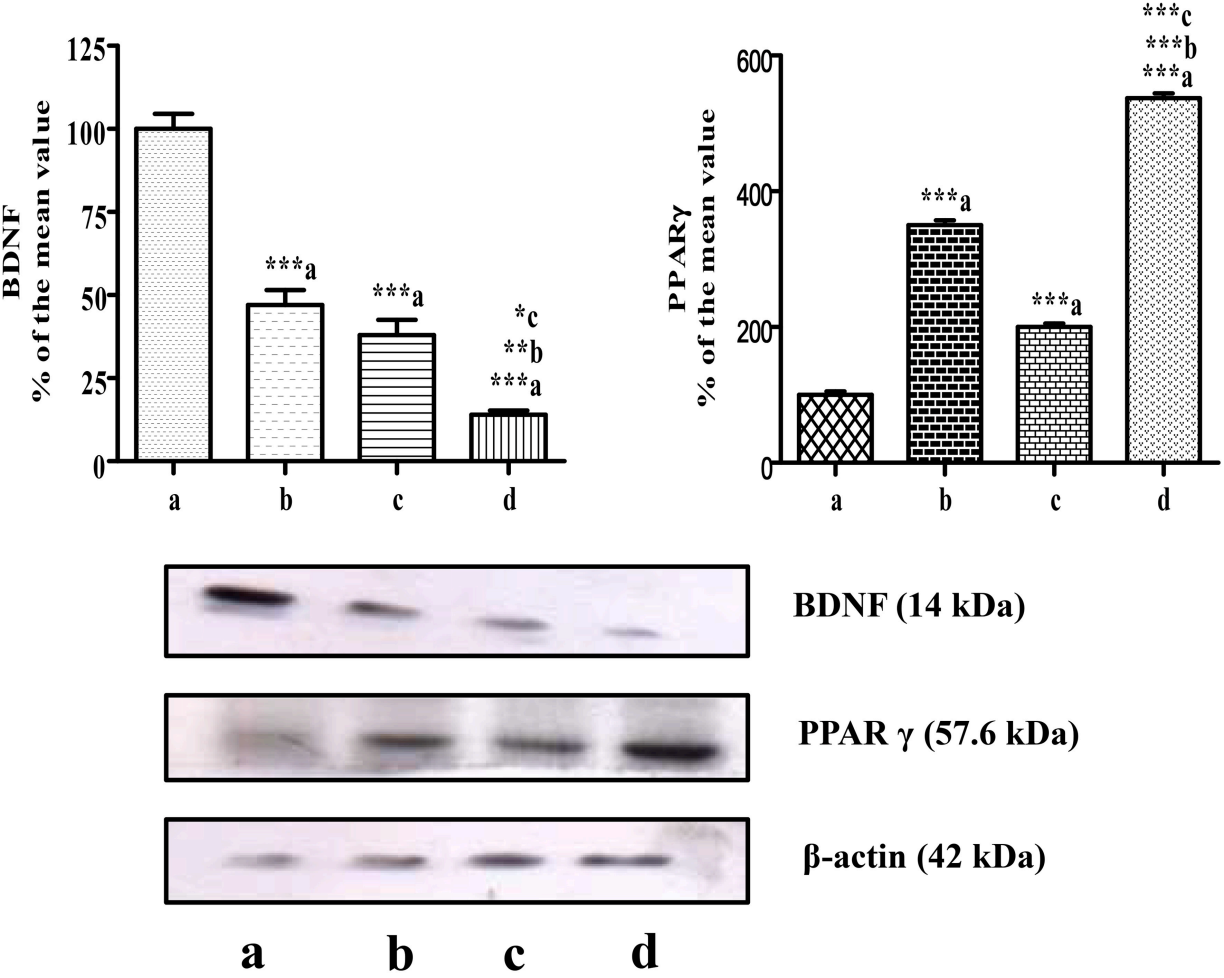
**Western blots of PPAR-γ and BDNF in mammary tumors**. Western blots of PPAR-γ and BDNF levels in mammary tumors after combined treatment of curcumin and cisplatin in breast cancer rats. Where lane a is breast cancer control, b is curcumin, c is cisplatin, and d is Curcumin + Cisplatin. Results were normalized with respect to actin. Similar results were obtained in three independent set of experiments. All values were expressed as mean ± SEM (*n* = 3). ^*^P < 0.05, ^**^P < 0.01, ^***^P < 0.001; a vs. breast cancer control & b vs. cisplatin.

The level of BDNF in mammary tumors was measured on the 5th day after the administration of CUR, CIS, and combination of CUR with CIS. The drug treated groups displayed significant reductions (*p* < 0.001) in circulating BDNF levels compared to control rats. Moreover, the combination of CUR with CIS showed a maximum reduction in the BDNF level when compared with CUR (*p* < 0.001) and CIS (*p* < 0.01) alone treated groups. Likewise, similar findings were observed in Western Blotting for quantification of BDNF supporting ELISA results (Figures 5, 6).

### Combined effect of CUR and CIS on PPAR-γ expression in mammary cancer rats

In this study, mammary cancer control rats showed low PPAR-γ expression, when compared with all drug-treated groups. However, we found higher PPAR-γ expression in CUR (*p* < 0.001) and CIS (*p* < 0.001) treated rats as compared to cancer control rats. Moreover, CUR pre-treatment for 5 days in CIS treated rats showed maximum expression of PPAR-γ when compared with CUR (*p* < 0.001) and CIS (*p* < 0.001) alone treated rats (Figure 6).

### Combined effect of CUR and CIS on renal histology in mammary cancer rats

**Figure 7:**
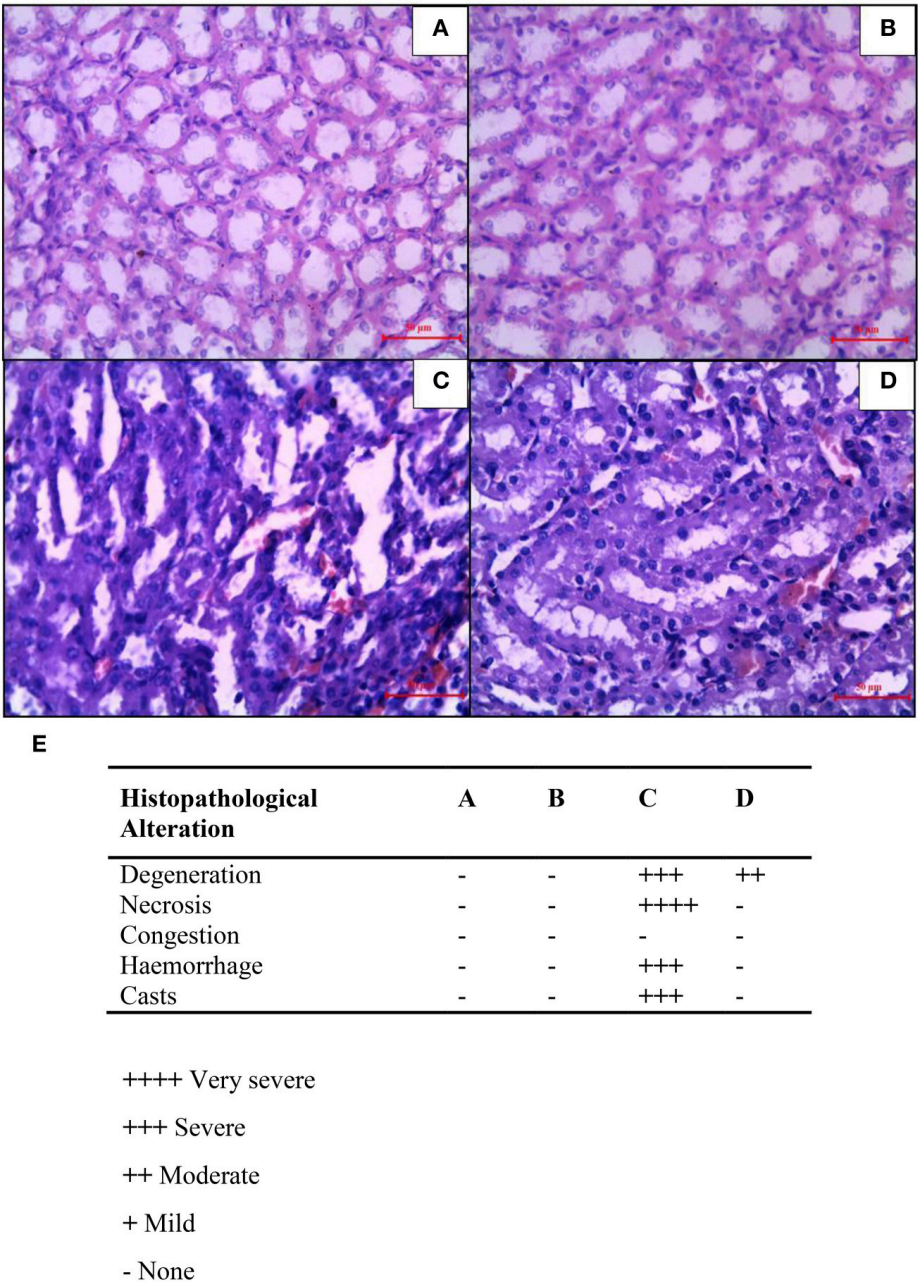
**Histopathological changes in the kidney after combined treatment of curcumin and cisplatin in breast cancer rats**. Transverse section of cancer control rat kidney **(A)**, kidney after treatment with curcumin **(B)**, cisplatin-treated **(C)**, and pre-treatment with curcumin **(D)**. Sections were stained with Mayer's hematoxylin counterstained with eosin and observed under magnification of 40X. Quantitative analysis of kidney histopathology **(E)** where; control rat kidney (A), curcumin treated kidney (B), cisplatin-treated kidney (C), and curcumin treated kidney (D). ++++, Very severe; +++, Severe; ++, Moderate; +, Mild; –, None.

In mammary cancer control rats, we observed intact renal tubules and glomeruli (Figure 7A). In addition, uniform tubules with a single layer of epithelium lining were observed in renal cortex in mammary cancer control rats. CIS treated rats revealed necrosis, protein cast, vacuolation, and desquamation of epithelial cells in renal tubules (Figure 7C). However, CUR pre-treatment for 5 days in CIS treated rats significantly protected the kidney architecture as compared to CIS treated rats (Figure 7D). CUR alone treated rats had no effect on renal histology (Figure 7B). Figure 7E represents the quantification of kidney histopathology.

### Combined effect of CUR and CIS on mammary tumor histology

**Figure 8:**
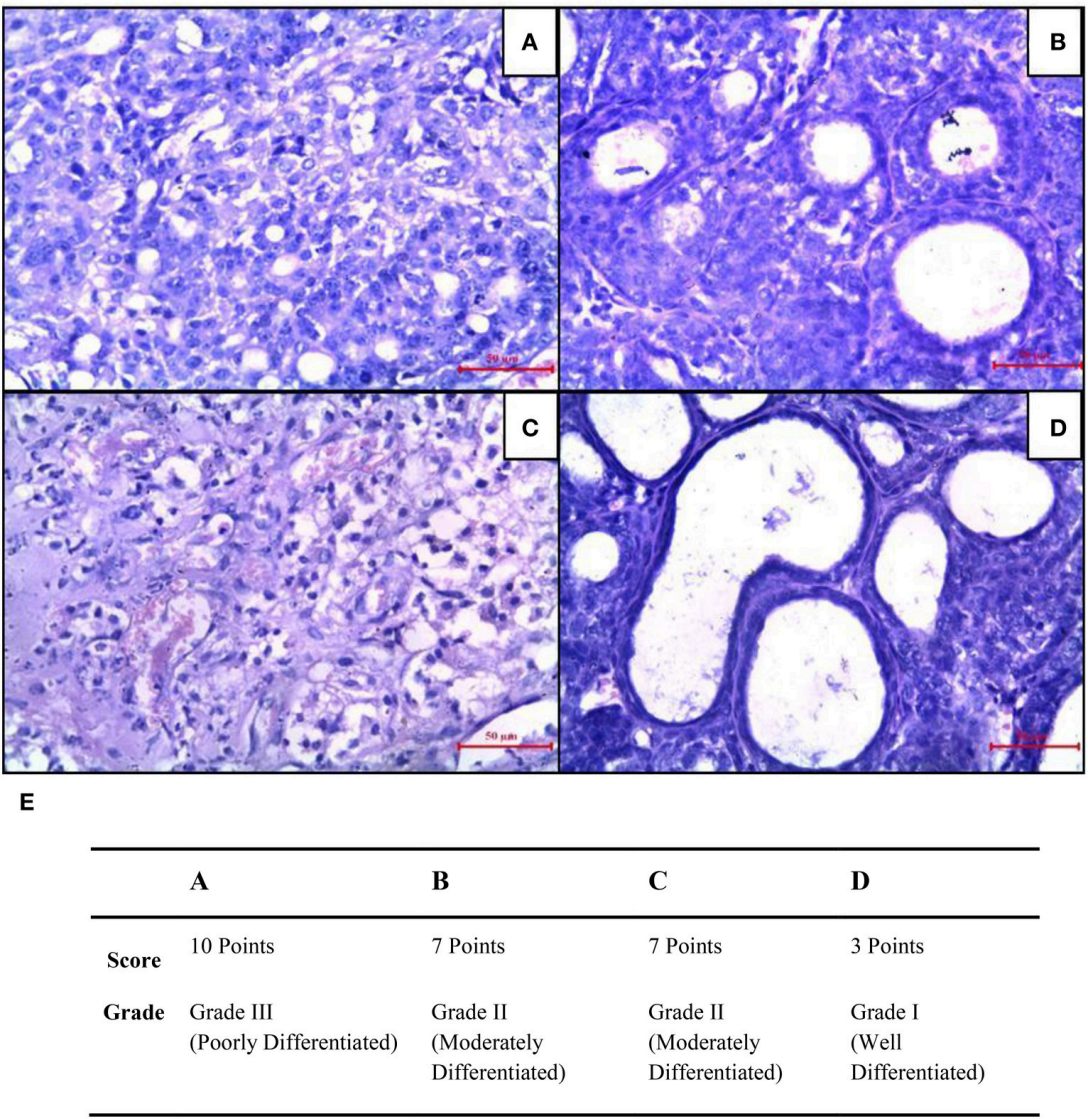
**Histopathological changes were seen in mammary tumors after combined treatment of curcumin and cisplatin. (A)** Breast cancer control group, **(B)** mammary tumor after treatment with curcumin: pronounced cell pleomorphism and a low grade of differentiation are demonstrated by multinucleated giant cells, **(C)** cisplatin treated mammary tumor with decreased cell density and higher level of fibrosis as sign of a therapeutic effect, **(D)** curcumin pre-treated mammary tumor after 5 days. The glandular structure as an indicator for a functional differentiation noticeable. **(E)** Quantitative analysis of histopathological studies in mammary tumor where, breast cancer control (A), curcumin treated mammary tumor (B), cisplatin-treated mammary tumor (C), and curcumin pretreated mammary tumor (D).

Mammary cancer control rats showed nuclear pleomorphism, abundant mitotic figures, and atypical mitotic figures (Figure 8A). CUR treated rats showed a low grade of differentiation which was demonstrated by giant multinucleated cells (Figure 8B). Decreased cell density and a higher level of fibrosis were observed in CIS treated animals (Figure 8C). However, CUR plus CIS treatment decreased nuclear pleomorphism along with a decrease in mitotic figures as well as atypical mitotic figures (Figure 8D), suggesting a combination of CUR plus CIS prevents tumor progression significantly. Figure 8E represents the quantification of mammary tumor histopathology.

## Discussion

The observation of the present study is that activation of PPAR-γ and inactivation of BDNF in mammary tissue inhibited the growth of breast cancer in rats. The cumulative treatment of CUR plus CIS augmented the expression of PPAR-γ while lessening the expression of BDNF in mammary tumors. The subsequent finding of this work is that CUR pre-treatment ablated the cisplatin-induced nephrotoxicity in breast cancer rats. CIS injection to tumor bearing rats resulted in kidney toxicity by means of inflammatory pathways. Mechanistically, CUR pre-treatment ameliorated the cisplatin-induced nephrotoxicity by inhibiting the pro-inflammatory cytokines like TNF-α, IL-6, IL-8, and augmenting anti-inflammatory cytokine (IL-10) in mammary tumor bearing rats. However, CUR did not affect the inflammatory responses in other groups (BCC, CUR).

In this work, the CIS injection significantly elevated renal pro-inflammatory cytokines in breast cancer rats. Conversely, CUR pre-treatment significantly reduced the pro-inflammatory cytokines and improved the anti-inflammatory cytokine in mammary tumor bearing rats. Our study outcomes paralleled with the findings of Kuhad and his colleagues, in which they have proved the renoprotective effect of curcumin in cisplatin-induced nephrotoxicity. They have studied low dose of CUR with different pre and post-treatment time schedule to check the renoprotective effect of CUR in CIS-induced nephrotoxicity through anti-inflammatory and antioxidant mechanisms (Kuhad et al., 2007). In the present work, we have used higher doses of curcumin and found that it safely and effectively suppressed CIS-induced renal inflammation in rats; subjects treated with CUR alone showed no specific side-effects or toxicity. Moreover, the effects of the inflammatory parameters were in full accord with the histopathological observations of this field.

The exact mechanisms underlying the suppression of nephrotoxicity by CUR are not fully revealed. The structure of CUR is comprised of a number of functional groups. The two aromatic phenol rings (rings A and B) are linked by two sets of α, β-unsaturated carbonyl groups that can react with glutathione and other nucleophiles. The 2 aryl methoxyl groups at the ortho position, the hydroxyl moiety as well as the conjugated β-diketone moieties of curcumin, which are conjugated, are other pharmacologically important structural features (Nabavi et al., 2014). The α, β-unsaturated diketone structural moieties of curcumin is responsible for heme oxygenase-1 and NFκB suppression (Rajasekaran, 2011). Sandur and his colleagues also reported that the α, β-unsaturated diketone moiety, in conjugation with phenolic rings, has a crucial role in NFκB activity (Sandur et al., 2007). Our results are in accordance with previous studies which reported that CUR down-regulates the transcription factor NFκB and suppresses various inflammatory mediators (Jobin et al., 1999; Kim et al., 2005). Ueki and colleagues have shown that CUR enhances CIS-induced nephrotoxicity by inhibiting renal inflammation in mice (Ueki et al., 2013). Tikoo and colleagues have shown that anti-inflammatory agents like rosiglitazone could prevent the CIS-induced nephrotoxicity (Tikoo et al., 2009b). In this study, we did not examine whether CUR inhibits NFκB activation or not. Further studies may be proposed to assess the effects of CUR administration on NFκB activation in CIS-induced nephrotoxicity in mammary gland cancer rats.

Side by side, we discovered that the combination of CUR sensitizes tumor cells to CIS. The compounding combination of the CUR with CIS shows a maximum decrease in the percentage tumor inhibition on 6th and 9th days as compared to treatment with CIS or CUR alone. The enhanced efficacy of the combination can either be due to the different mechanism of these drugs (improved expression of PPAR-γ and reduced expression of BDNF) or the chemosensitizing effect of CUR.

On that point are increasing evidence demonstrating that pharmacological activation of PPAR-γ results in anticancer activity in experimental models of breast cancer (Rubin et al., 2000; Qin et al., 2003). The CUR has been shown to protect against chemically induced breast cancer (Kumar et al., 2015). Still, it is unclear whether CUR protects from breast cancer by activation of PPAR-γ or not. Present data have indicated a significant gain in both aspect and action of PPAR-γ in the breast cancer tissue by pre-treatment of CUR, suggesting an agonistic effect of CUR on PPAR-γ. Similarly, in our earlier study, we had disclosed that PPAR-γ agonist, rosiglitazone, amplified PPAR-γ expression in DMBA induced breast cancer (Tikoo et al., 2009b). Consistent with the previous work, the present data demonstrated that activation of the PPAR-γ by curcumin was crucial for all facets of curcumin's anticancer activity in rats.

In this study, we observed that CUR pre-treatment for 5 days in CIS treated rats increased the PPAR-γ expression in mammary tumors and hence highest anticancer activity amongst the entire study drug-treated groups and this may be possibly responsible for the decreased proliferation and enhanced apoptosis of mammary cancer cells. Moreover, only CUR treated rats showed high expression of PPAR-γ when compared with CIS treated groups. This difference was attributed to the agonistic action as well as 5 doses of CUR as compared to a single dose of CIS and no agonistic action. CUR and CIS treated rats exhibited a maximum reduction in tumor mass and substantial improvement in tumor morphology, further strengthening the above resolution. The finding of this study was also in hormony with our previous study (Tikoo et al., 2009b) in which we had tried rosiglitazone as a PPAR-γ agonist in combination with CIS as an anticancer cancer agent in DMBA induced breast cancer in rats. In that study, rosiglitazone ameliorated the CIS-induced renotoxicity and in chorus synergize the anticancer cancer activity (through the PPAR-γ pathway). The current study also indicated a good function of PPAR-γ specific ligands in the chemoprevention of mammary carcinogenesis.

The stimulus of resistance to apoptosis by BDNF suggests their possible value as therapeutic targets in breast cancer, that provide new directions for the invention of innovative strategies based on neurotrophin inhibition. A previous study claimed that higher level of BDNF is significantly related to breast cancer development and its prohibition leads to reduced tumor cell survival (Vanhecke et al., 2011). It was further reported that the mammary cancer specimens have a high level of BDNF as compared to normal tissue of human subjects (Patani et al., 2011). Likewise, in the present work, we also noted higher levels of BDNF in breast cancer tissue. CUR pre-treatment for 5 days followed by single injection of CIS significantly reduced BDNF expression and inhibited progression of mammary cancer. Interestingly, CUR plus CIS combination presented maximum fall in BDNF level as compared to CUR or CIS treated breast cancer rats.

In conclusion, our data suggest that curcumin pre-treatment, along with cisplatin, potentates the antineoplastic activity of the CIS, as well as attenuates its renotoxicity. Thus, this combination could lead to the development of a novel therapeutic approach with high antineoplastic activity and low renotoxicity. The combination of CUR and CIS may have profound clinical implications in breast cancer treatment, yet further studies may be advised to ensure the utility of this permutation in different breast cancer models.

## Ethics statement

This study was carried out in accordance with the Institutional Animal Ethical Committee of Gauhati Medical College and Hospital, which approved the protocol.

## Author contributions

PK: Designing of Hypothesis, Literature Reviewing, Research Work, Statistically analysis, Manuscript Editing, Manuscript proofreading, Approval for the final version. CB: Designing of Hypothesis, Literature Reviewing, Manuscript Editing, Manuscript proofreading. Approval for the final version. KS: Literature Reviewing, Research Work, Statistically analysis, Manuscript Editing, Manuscript proofreading. RS: Designing of Hypothesis, Research Work, Statistically analysis, Manuscript proofreading, Approval for the final version.

### Conflict of interest statement

The authors declare that the research was conducted in the absence of any commercial or financial relationships that could be construed as a potential conflict of interest. The reviewer DS and handling Editor declared their shared affiliation, and the handling Editor states that the process nevertheless met the standards of a fair and objective review.
